# Accuracy of ICD-10 Codes for Surveillance of *Clostridium difficile* Infections, France

**DOI:** 10.3201/eid1806.111188

**Published:** 2012-06

**Authors:** Gabrielle Jones, Namik Taright, Pierre Yves Boelle, Jeanne Marty, Valérie Lalande, Catherine Eckert, Frédéric Barbut

**Affiliations:** Sainte-Antoine Hospital–Assistance Publique Hôpitaux de Paris, Paris, France

**Keywords:** Clostridium difficile, International Classification of Diseases, ICD-10 codes, surveillance, epidemiology, bacteria, France

## Abstract

The sensitivity and specificity of surveillance for *Clostridium difficile* infections according to International Classification of Diseases, 10th revision, codes were compared with laboratory results as standard. Sensitivity was 35.6%; specificity was 99.9%. Concordance between the 2 methods was moderate. Surveillance based on ICD-10 codes underestimated the rate based on laboratory results.

*Clostridium difficile* causes 15%–25% of diarrhea after antimicrobial drug therapy and is the leading cause of nosocomial diarrhea in adults (*1*). Studies in the United States, Canada, and Europe have documented the increased rate and severity of *C. difficile* infections highlighting the need for efficient and accurate methods of surveillance (*2**–**7*). The use of International Classification of Diseases (ICD) codes for surveillance of *C. difficile* infections has been studied in the United States and in Singapore and showed discordant results (*8**–**12*). Our objective was to compare the sensitivity and specificity of surveillance for *C. difficile* infections on the basis of ICD, 10th revision (ICD-10), codes with surveillance based on laboratory results.

## The Study

The study was conducted at Saint-Antoine Hospital, a 750-bed university-affiliated public hospital in Paris, France. The study population comprised all patients hospitalized during January 1, 2000–December 31, 2010. *C. difficile* testing was performed only on unformed fecal samples of patients clinically suspected to have *C. difficile* infection. Laboratory diagnosis of *C. difficile* infection did not vary during the study period and was based on the stool cytotoxicity assay coupled with the toxigenic culture. A bacteriologic case of *C. difficile* infection was defined as a positive cytotoxicity assay result and/or a positive toxigenic culture.

Data were collected retrospectively from the electronic discharge summaries (French medico-administrative database) and from the hospital microbiology laboratory. All patients with a positive laboratory result for *C. difficile* (Bact+) and/or the ICD-10 discharge code for *C. difficile* infection, A04.7, as principal or associated diagnosis (ICD10+), were identified. For patients with multiple laboratory results during the same hospitalization, we used only the initial result.

We classified cases as concordant (Bact+/ICD10+) or discordant (Bact+/ICD10– or Bact–/ICD10+). Bact+/ICD10– discordant cases were compared with concordant cases to identify factors predictive of missing codes. Medical records were reviewed for Bact–/ICD10+ case-patients.

Statistical analysis included κ, χ^2^, and the Mann-Whitney U test. We used the Spearman test to measure the correlation between the 2 methods for yearly incidence of *C. difficile* infection. Data were analyzed with Epi Info version 6.01 (Centers for Disease Control and Prevention, Atlanta, GA, USA), GraphPad Prism version 4.03 (GraphPad Software, Inc., La Jolla, CA, USA) and R version 2.0 (R Foundation for Statistical Computing, Vienna, Austria).

**Figure 1:**
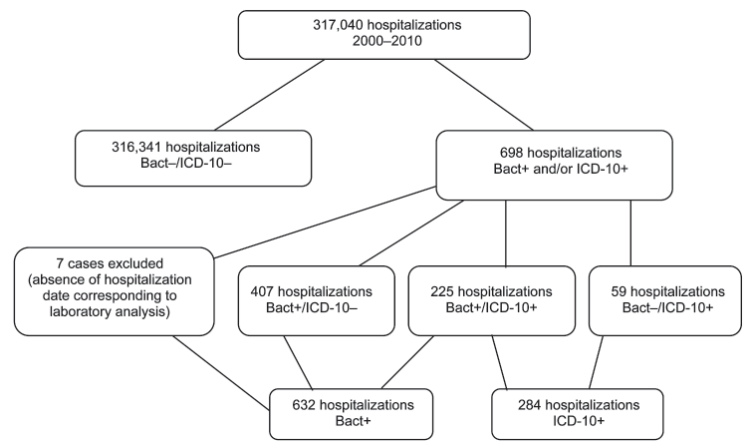
Flowchart of *Clostridium difficile* infections case classifications for patients admitted to Saint-Antoine Hospital, Paris, France, 2000–2010. Bact+, positive laboratory result for *C. difficile*; ICD10+, International Classification of Diseases, 10th Revision, discharge code for *C. difficile* infection, A04.7, as principal or associated diagnosis.

During 2000–2010, of 317,040 hospitalizations, laboratory results and/or the ICD-10 code for *C. difficile* infection were positive for 698 (Figure 1). Sensitivity of the ICD-10 code, with laboratory results as the standard, was 35.6% (95% CI 31.9%–39.5%), and specificity was 99.9% (95% CI 99.9%–100.0%). The positive and negative predictive values were 79.2% (95% CI 73.9%–83.7%) and 99.9% (95% CI 99.8%–99.9%), respectively (Table). The sensitivity of ICD-10 codes varied among hospital wards. For wards with >50 cases of *C. difficile* infections during 2000–2010, sensitivity ranged from 14% to 71.6%. Average sensitivity increased from 26% for 2000–2005 to 39% for 2006–2010 (p = 0.02). Overall, concordance between the 2 methods was moderate (κ = 0.49, p<0.0001).

**Table:** Sensitivity, specificity, and positive and negative predictive values of ICD-10 codes for *Clostridium difficile* infection, Saint-Antoine Hospital, Paris, France, 2000–2010*

Classification	Bact+	Bact–	Total
ICD-10+	225	59	284
ICD-10–	407	316,342	316,749
Total	632	316,401	317,033

*Sensitivity 35.6%, specificity 99.9%, positive predictive value 79.2%, and negative predictive value 99.9%. Bact+, positive result for *C. difficile*; Bact–, negative result for *C. difficle*; ICD-10, International Classification of Diseases, 10th Revision.

**Figure 2:**
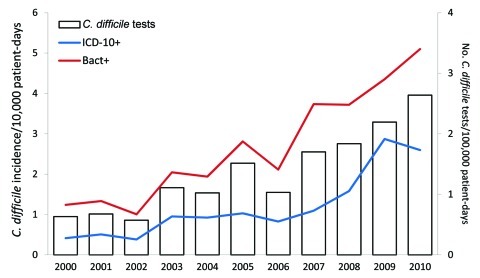
Incidence of *Clostridium difficile* infections by surveillance method and number of *Clostridium difficile* tests, Saint-Antoine Hospital, Paris, France, 2000–2010. Bact+, positive laboratory result for *C. difficile*; ICD10+, International Classification of Diseases, 10th Revision, discharge code for *C. difficile* infection, A04.7, as principal or associated diagnosis.

The incidence of *C. difficile* infection determined by ICD-10 codes underestimated the incidence determined by laboratory results. The relationship between methods for yearly incidence during the 11-year period was strong (Spearman correlation coefficient r = 0.95, 95% CI 0.81–0.98, p<0.0001). The rate of *C. difficile* infection by ICD-10 codes and laboratory results increased during 2000–2010 (Figure 2). The incidence of *C. difficile* infection also increased across all age groups. During 2000–2010, incidence increased by a factor of 3.3 for patients 15–44 years of age, by 2.9 for patients 45–64 years of age, and by 4.2 for patients >65 years of age.

Concordant cases (Bact+/ICD10+) and discordant cases (Bact+/ICD10–) did not differ significantly by mean age, sex, or proportion of patients >65 years of age. Diagnosis by positive toxigenic culture (with negative stool cytotoxicity assay result) was predictive for missing ICD-10 codes (χ^2^ = 19.22, p<0.0001), as was sample collection within 48 hours before discharge (χ^2^ = 16.57, p<0.0001). Patients with concordant results were more likely than patients with discordant results to have sample collection within 48 hours after admission (χ^2^ = 23.7, p<0.0001).

Review of medical records was possible for 34 (58%) of 59 discordant cases Bact–/ICD10+. Potential explanations for coding in the absence of a positive laboratory result included diagnosis outside the hospital (8 cases), positive result for a nontoxigenic strain of *C. difficile* (7 cases), diagnosis by endoscopy (6 cases), strong clinical suspicion of disease in patients with a history of *C. difficile* infection but no positive laboratory result (5 cases), and initial positive result subsequently corrected to a negative result by the laboratory (2 cases). No explanation could be found for the ICD-10 code in 6 cases: 5 had a negative laboratory result for *C. difficile* in the medical record, and 1 had no record of clinical suspicion or fecal sample collection.

## Conclusions

This study covers an 11-year period and provides a large study population and more comprehensive analysis of the performance of ICD-10 codes. Our results indicate that surveillance for *C. difficile* infections based on ICD-10 codes underestimates the rate of *C. difficile* infections based on microbiological findings at Saint-Antoine Hospital. Even though trends in *C. difficile* infections incidence for the 2 methods correlated strongly, concordance was moderate.

The sensitivity of ICD-10 codes in this study is inferior to values previously reported in the United States (71%–78%) and in Singapore (49.6%) (*8**–**11*). Poor sensitivity and variability among wards could be attributed to differences in awareness by health care professionals of *C. difficile* infections and to differences in coding practices. At Saint-Antoine Hospital, coding is performed by physicians with limited training, not by trained medical coders. Therefore, the quality of coding can vary from 1 physician to another and among wards. In addition,, differences in sensitivity could be explained by changes in hospital financing. As of 2006, funding for hospitals in France has been connected to coding through Activity Based Payment (*13*). Comparison of average sensitivity before and after 2006 showed an overall increase, indicating that coding practices might improve with time as hospitals adapt to this system.

Our finding that sample collection within 48 hours before hospital discharge was predictive of missing ICD-10 codes is consistent with findings from previous studies and suggests that results obtained after patient discharge are less frequently coded (*8**,**9**,**12*). Diagnosis by toxigenic culture was also significantly associated with missing ICD-10 codes. The toxigenic culture is a long test requiring up to several days for results. At Saint-Antoine Hospital, results are provided at each step of analysis (stool cytotoxicity assay in 24 h, culture in 48 h, toxigenic culture within 5 d), which might introduce misinterpretation of preliminary results before the final comprehensive result.

Analysis of medical records for patients coded for *C. difficile* infections but lacking a positive laboratory result suggested several potential explanations for coding. Diagnoses made outside the hospital and those made by endoscopy are coded, indicating that cases diagnosed by methods other than in-hospital laboratory testing are captured by ICD-10 codes.

This study was limited to a single institution, and our findings might not necessarily apply to other institutions in France. The sensitivity of ICD-10 codes can be highly variable, and this method should be validated in different health care settings before being used for surveillance.

Use of ICD-10 codes underestimates the incidence of *C. difficile* infections compared with microbiological data. However, it may be an effective indicator for monitoring general trends in the rate of *C. difficile* infection.
